# Pathogenic variant detection rate varies considerably in male breast cancer families and sporadic cases: minimal additional contribution beyond *BRCA2, BRCA1* and *CHEK2*

**DOI:** 10.1136/jmg-2023-109826

**Published:** 2024-04-12

**Authors:** D Gareth Evans, George J Burghel, Sacha J Howell, Sarah Pugh, Claire Forde, Anthony Howell, Fiona Lalloo, Emma Roisin Woodward

**Affiliations:** 1 Genomic Medicine, MAHSC, Manchester, UK; 2 Genomic Diagnostic Laboratory, Manchester University NHS Foundation Trust, Manchester, UK; 3 Wythenshawe Hospital Manchester Universities Foundation Trust, Wythenshawe, UK; 4 Manchester Academic Health Science Centre, Manchester, UK; 5 Manchester University Hospitals NHS Foundation Trust, Manchester, UK; 6 Clinical Genetics Service, Central Manchester University Hospitals NHS Foundation Trust, Manchester, UK; 7 Prevent Breast Cancer Centre, Manchester, UK; 8 Clinical Genetics Service, Manchester Centre for Genomic Medicine, Central Manchester University Hospitals NHS Foundation Trust, Manchester, UK; 9 Manchester Centre for Genomic Medicine, Central Manchester NHS Foundation Trust, Manchester, UK

**Keywords:** Molecular Epidemiology, Men's Health, Human Genetics, Genetic Testing, Genetic Counseling

## Abstract

**Background:**

Male breast cancer (MBC) affects around 1 in 1000 men and is known to have a higher underlying component of high and moderate risk gene pathogenic variants (PVs) than female breast cancer, particularly in *BRCA2*. However, most studies only report overall detection rates without assessing detailed family history.

**Methods:**

We reviewed germline testing in 204 families including at least one MBC for *BRCA1*, *BRCA2*, *CHEK2* c.1100DelC and an extended panel in 93 of these families. Individuals had MBC (n=118), female breast cancer (FBC)(n=80), ovarian cancer (n=3) or prostate cancer-(n=3). Prior probability of having a *BRCA1/2* PV was assessed using the Manchester Scoring System (MSS).

**Results:**

In the 204 families, *BRCA2* was the major contributor, with 51 (25%) having PVs, followed by *BRCA1* and *CHEK2,* with five each (2.45%) but no additional PVs identified, including in families with high genetic likelihood on MSS. Detection rates were 85.7% (12/14) in MSS ≥40 and 65.5% with MSS 30–39 but only 12.8% (6/47) for sporadic breast cancer. PV rates were low and divided equally between *BRCA1/2* and *CHEK2.*

**Conclusion:**

As expected, *BRCA2* PVs predominate in MBC families with rates 10-fold those in *CHEK2* and *BRCA1*. The MSS is an effective tool in assessing the likelihood of *BRCA1/2* PVs.

## Introduction

Since the identification of the *BRCA1* and *BRCA2* genes, germline pathogenic and likely pathogenic variants, herein collectively termed PVs, have been associated with increased risks of breast cancer in both men and women. The cumulative breast cancer risk at age 80 years is about 72% and 69% for female *BRCA1* and *BRCA2* PV carriers, respectively, whereas it is around 0.4% and 4% for males, respectively.^1 2^ More recently, extended gene panels that include additional breast cancer genes have been employed. Two recent large case control studies CARRIERS in the USA^3^ and BRIDGES mainly in Europe^4^ identified six additional genes with actionable increased risks of breast cancer with ORs of around twofold to threefold (*RAD51C*, *RAD51D*, *BARD1*, *ATM*, *CHEK2*) and one at fourfold to fivefold (*PALB2*). A number of studies have assessed an extended panel in large series of male breast cancer (MBC) but these have not assessed detection rates by strength of family history.^5–8^ We have updated our work on MBC cases and MBC families^9^ to include more testing in sporadic cases of MBC with no personal or family history of the relevant cancers and increased the overall sample size from 68 to 204.

## Patients and methods

Families with breast cancer from the North-West of England (population~5.5 million) have been tested for *BRCA1/2* PVs since 1996 in the Manchester Centre for Genomic Medicine. People who attend the specialist genetic clinics in Manchester with a personal or family history of breast cancer have a detailed family tree elicited with all first-degree, second-degree and, if possible, third-degree relatives recorded including age and details of related cancers (breast, ovarian, prostate, pancreas).

Families with at least one confirmed MBC were eligible for genetic testing as long as the MBC case or a first degrre relative of the MBC case with a relevant cancer was tested. Non-male breast cancer cases were eligible as long as they had developed breast, ovarian, or prostate cancer and were a first degree relative of a MBC case. Sporadic MBC were defined as men affected with breast cancer with 1st or 2nd degree relatives without a personal history of breast, ovarian, prostate or pancreatic cancer. Samples from individuals with a relevant cancer from 204 apparently unrelated families with a confirmed MBC underwent genetic testing. The 204 comprised index individuals with MBC (n=118), female breast cancer (FBC) (n=80), ovarian cancer (n=3) and prostate cancer (n=3).

DNA was extracted from lymphocytes and tested using a combination of targeted sequencing and multiplex ligation-dependent probe amplification for all genes assessed.^10^ All participants underwent testing of *BRCA1*, *BRCA2* and *CHEK2* c.1100delC. Additional testing was performed in 93 cases with an extended panel including full screening of *CHEK2*, *ATM*, *PALB2*, *RAD51C and RAD51D* for samples since 2020 (n=51) and retrospectively for samples with high Manchester scores and negative *BRCA1/2* testing (n=42). Between 2018 and 2020, a bespoke in-house Manchester panel could be ordered which included *CDH1*, *BARD1* and *BRIP1*. Additional research testing was carried out on *BRIP1* and for lobular breast cancer cases for *CDH1*.

The Manchester Scoring System (MSS) was employed to evaluate the likelihood of a PV in *BRCA1* and *BRCA2* combined.^11^ The MSS provides a points score to each cancer in the individual and direct family lineage and adjusts for tumour pathology including a score of minus 6 points for HER2+ breast cancer and plus 6 for grade 3 triple negative breast cancer (online supplemental table 1).^11^ An MSS of 15–19 equates to a 10% likelihood of a *BRCA1/2* PV, and MSS 20–24 to a 20% likelihood, with an MSS≥40 equivalent to a >75% likelihood.^11^

10.1136/jmg-2023-109826.supp1 Supplementary data

Statistical analyses using χ^2^ Fisher’s exact tests were conducted for two by two comparisons. Median and IQR were calculated in Excel.

## Results

Samples from individuals with a relevant cancer from 204 apparently unrelated families with a confirmed MBC underwent genetic testing. PVs in *BRCA2* were identified in 51 out of 204 (25%) individuals overall (19/118 (16%) MBC, 30/80 (37.5%) FBC, 1/3 prostate cancer, 1/3 ovarian cancer). The mean MSS for MBC was 17.1 (median=15; IQR=12–19) compared with 27.5 (median=25; IQR=19–31.5) for female cases. Excluding the 47 sporadic MBCs, the detection rate in MBC was 18 out of 71 (25%), with a mean MSS of 21.4 (median=19; IQR=16–25). For the sporadic MBC cases, only 3 out of 47 had PVs in *BRCA1/2* with the same proportion having a *CHEK2* c.1100delC PV. Surprisingly, there was no effect of early age at onset for *BRCA2* with both PVs in sporadic cases occurring at age >60, with one MBC excluded from the sporadic set at age 66 with *BRCA2* having developed prostate cancer before testing at age 68. The mean age for MBC case PV carriers by gene were 63.8 for *BRCA2*, 60.7 for *BRCA1* and 52 for *CHEK2* cases. The mean age of those without PVs was 60.6 years. No PVs were detected in wider panel testing to include intragenic coding variants of *ATM*, *PALB2*, *RAD51C* and *RAD51D*.

The MSS was highly predictive of a *BRCA1/2* PV (table 1). In keeping with the published thresholds, an MSS <15 (there is a minimum score of 10 with a MBC >59) demonstrated a detection rate below 10% at 9.5% (6/63). The 15–19 range identified 10 PVs (19.6%) with 12/14 (85.7%) of those with an MSS ≥40 having a *BRCA1/2* PV. While increasing MSS was associated with ever increasing PV rates, especially for *BRCA2,* this was not predictive for *CHEK2*. No PVs were identified in 93 cases with full gene panel testing including 11 out of 12 with MSS ≥30 who tested negative for *BRCA1/2*. The only non-c.1100delC *CHEK2* variant (c.1263delT) was in an MBC case with a very strong family history of FBC and an MSS of 39. Additional genes tested with a definite link to breast cancer included *CDH1* (n=29) and *BARD1* (n=19) testing negative in addition to 72 samples testing negative for *BRIP1*.

**Table 1:** PV detection rates by Manchester Scoring System and age of sporadic male breast cancer

MSS	Number tested	*BRCA1 PVs* (%)	*BRCA2 PVs* (%)	*BRCA1/2* (%)	*CHEK2*	%	Other genes tested	Positive
<15	63	1 (1.59%)	5 (7.94%)	9.52%	3	4.76%	43	0
15–19	51	1 (1.96%)	9 (17.65%)	19.61%	0	0.00%	20	0
20–29	50	0 (0%)	12 (24.0%)	24.00%	1	2.00%	17	0
30–39	26	2 (7.69%)	14 (53.85%)	61.54%	1	3.85%	9	0
≥40	14	1 (7.14%)	11 (78.57%)	85.71%	0	0.00%	4	0
Total	204	5 (2.45%)	51 (25.0%)	27.45%	5	2.45%	93	0
>29 no BRCA1/2	12	N/a	N/a		1	8.33%	11	0
**Sporadic MBC**								
<60	22	1 (4.55%)	0 (0%)	4.55%	2	9.09%	15	0
≥60	25	0 (0%)	2 (8.0%)	8.00%	1	4.00%	23	0
Total	47	1 (2.13%)	2 (4.26%)	6.38%	3	6.38%	38	0

PV, pathogenic variant.

Full receptor status was available for 13 *BRCA2* MBC and all were ER+HER2, with 5 out of 13 grade 3 (8 grade 2). Similarly, all *CHEK2*-associated MBC were ER+HER2 and 2 out of 4 grade 3. Both the *BRCA1* PV heterozygotes with full pathology had ER+HER2- cancers. Of the MBCs with no PV and full pathology, 11 out of 40 had grade 3 tumours (1x grade 1) and all were ER+HER2-.

In total, 17 non-index cases with MBC have been tested in *BRCA1/2* PV families, with only one testing negative, an 83 year old, for *BRCA1*.

## Discussion

In the current study, we have shown a high detection rate for *BRCA2* PVs with a detection rate 10-fold higher than for *BRCA1,* consistent with the 10-fold higher lifetime risk in *BRCA2* PV heterozygotes.^1 2^ Interestingly, age of onset of MBC was not predictive of a *BRCA2* PV. This is a similar finding to a large German cohort where 142 *BRCA2* PVs were found in cases with a mean age at onset of 62 years compared with 60 years of cases without a PV.^6^ The overall detection rate was similar to this study at 23% but is higher than ours for the MBC cases themselves (16%). Similarly, the *BRCA1* rate of 4.6% was almost double than the 3 out of 118 (2.5%) in the current study. The German study had a lower proportion of sporadic MBC 146/614 (23.8%) compared with 47/118 (39.6%) in the current study likely reflecting a stronger selection bias. However, the *BRCA2* detection rate was also around half in the current study (4.3%) compared with 14/146 (9.6%).^6^ This may reflect a lower *BRCA2* population frequency in the UK. The frequency of *CHEK2* PVs was 3.2% (11/340) in the German study in *BRCA1/2* negative samples compared with 4/92 (4.3%) MBC cases in the current study. After *CHEK2*, *PALB2* was the most frequently involved, with 6 out of 340 compared with none in our case set despite *PALB2* having a potential founder effect in our region.^12^ The additional panel beyond *CHEK2* of definitive breast cancer genes only identified a further 9 out of 340 (2.6%) in the German study with *ATM* the most frequent (n=6). There was no detailed breakdown of degree of family history in the German study and they did not include prostate and pancreatic cancer in their analysis.

A large Italian study, the most recent of several from the consortium, tested 767 *BRCA1/2*-negative MBCs and 1349 male controls for an extended panel of 50 genes.^5^ Although their headline rate was 4.8% (n=37), this dropped to only 18 (2.3%) if only definitive breast cancer genes^3 4^ were included (2 PVs were also found in *NF1* which is also associated outside case control studies but is not usually in breast cancer gene panels). PV carriers were more likely to have had a personal (p=0.03) and family (p=0.02) history of cancers not limited to breast cancer. They found that *PALB2* PVs were associated with a sevenfold increased MBC risk (OR=7.28, 95% CI=1.17–45.52; p=0.034), and *ATM* PVs with a fivefold increased MBC risk (OR=4.79, 95% CI=1.12–20.56; p=0.035). As no *CHEK2* variants were found in controls, they did not report significance for this gene. However, *CHEK2* was significant by our estimate on χ^2^ testing: 3/767 vs 0/1349 (p=0.04). A small Finnish study also found a significant risk from the *CHEK2* c.1100delC PV which was found in 4 out of 84 patients (5.9%), significantly more frequently than in the controls (OR=4.47, 95% CI=1.51–13.18, p=0.019).^7^ Finally, a testing study of panel testing from Ambry genetics of 715 MBCs found a headline PV detection rate of 18.1% for 16 breast cancer susceptibility genes and with no prior *BRCA1/2* testing (n=480). *BRCA2* and *CHEK2* were the most frequently identified (11.0% and 4.1%), with *BRCA2* (OR=13.9; p=1.92×10^-16^), *CHEK2* (OR=3.7; p=6.24×10^-24^) and *PALB2* (OR=6.6, p=0.01).^8^ Apart from six *ATM* variants (1%), the additional detection rate in those with no prior screen was only 0.8% with just two *PALB2* PVs and one *RAD51D*. There was no detailed breakdown of family history.

The current study has some limitations. It is not as large as some previous studies but includes testing of families where MBC occurred in a first-degree relative. Not all individuals that tested negative for *BRCA1/2* have had an extended panel, although 92% of those with an MSS ≥30 have been tested, revealing just one non*-CHEK2* c.1100delC variant. Pathology was also not complete but reflects the historical nature of some MBC cases that were diagnosed prior to routine testing of receptor status. The main strength of the study is the extensive collection of family history. This is the first study to our knowledge to validate a *BRCA1/2* likelihood algorithm specifically in MBC families. The extended panel testing was also not sufficiently large to rule out detection rates in non-*BRCA1/2/CHEK2* genes at a rate below 2%.

In summary, the current study has demonstrated a *BRCA2* PV rate in MBC families 10-fold that of *BRCA1,* with *CHEK2* being the only other gene in which PVs were identified. The MSS has shown good validation as a predictor of *BRCA1/2* likelihood. The detection rate of validated breast cancer genes beyond *BRCA1/2 and CHEK2* from the current study, and the literature, is low at ~2%.
