# Supplementary material for: Pathogenic variant detection rate varies considerably in male breast cancer families and sporadic cases: minimal additional contribution beyond BRCA2, BRCA1 and CHEK2
Source: J Med Genet. 2024 Apr 12;61(9):853–5. doi: 10.1136/jmg-2023-109826 (PMC11420751; doi:10.1136/jmg-2023-109826)
Supplement: Supplementary data [file jmg-2023-109826supp001.pdf]

Supplementary table 1: Pathology adjusted Manchester score criteria

**Cancer and age at diagnosis -Score each cancer in a direct lineage in the family add BRCA1 & BRCA2 for combined score**	***BRCA1***	***BRCA2***
**FBC <30**	**6**	**5**
**FBC 30-39**	**4**	**4**
**FBC 40-49**	**3**	**3**
**FBC 50-59**	**2**	**2**
**FBC >59**	**1**	**1**
**MBC <60**	**5**	**8**
**MBC >60**	**5**	**5**
**Ovary <60**	**8**	**5**
**Ovary >60**	**5**	**5**
**Pancreas**	**0**	**1**
**Prostate <60**	**0**	**2**
**Prostate >60**	**0**	**1**

**Breast cancer**	**BRCA1 adjustment**	**BRCA2 adjustment**
HER2 positive	-6 **+/- grade ER**	0
Lobular	-2 +/- ER status	0
DCIS only	-2 +/- ER status	0
LCIS only	-4 **no other adjustment**	0
Grade 1 IDC	-2 +/- ER status	0
Grade 2 IDC	0 +/- ER status	0
Grade 3 IDC	+2 +/- ER status	0
ER positive	-1 +/- grade	0
ER negative	+1 +/- grade	0
Triple negative	+4 +/- grade ie grade 3 TNT= +6	0
**Ovarian cancer**		
High grade serous <60	+2	0
Epithelial (endometrioid, serous, clear cell)<br>granulosa cell	none	none

Mucinous, borderline or germ cell tumours (except granulosa cell)	no score	no score
**Adopted**		
no known status in birth family	+2	+2

Pathology adjustment

FBC = Female Breast Cancer
MBC = Male Breast Cancer
Pancreas any gender
